# Linear association between the systemic immune-inflammation index and all-cause mortality in patients with interstitial lung disease: a retrospective cohort study

**DOI:** 10.3389/fmed.2026.1795802

**Published:** 2026-04-07

**Authors:** Haoran Chen, Minhua Shi, Yong Yu, Zengli Zhang, Ziyan Du

**Affiliations:** Department of Respiratory and Critical Care Medicine, The Second Affiliated Hospital of Soochow University, Suzhou, China

**Keywords:** all-cause mortality, cohort study, interstitial lung disease, prognosis, systemic immune-inflammation index

## Abstract

**Objective:**

To investigate the association between the systemic immune-inflammation index (SII) and all-cause mortality in patients with interstitial lung disease (ILD).

**Methods:**

This retrospective cohort study included 366 patients with ILD. SII was calculated using peripheral blood counts and analyzed as both a continuous and categorical variable based on the optimal cutoff value determined by receiver operating characteristic (ROC) analysis. Kaplan–Meier survival curves were used to compare survival between SII groups. Univariable and multivariable Cox proportional hazards models were applied to evaluate the association between SII and all-cause mortality. Restricted cubic spline (RCS) analysis was performed to assess the dose–response relationship. Subgroup analyses were conducted to examine the robustness of the association.

**Results:**

Over a median follow-up of 20.6 months, the primary outcome of all-cause mortality occurred in 91 patients (24.9%). The median SII was significantly higher in deceased patients compared with survivors (1471.14 vs. 1017.21). ROC analysis showed a statistically significant discriminatory ability of SII for mortality prediction (AUC = 0.658, 95% CI 0.594–0.723). Kaplan–Meier analysis demonstrated significantly lower survival in patients with high SII (log-rank *p* < 0.001). In multivariable Cox models, higher SII remained independently associated with increased all-cause mortality, showing consistent associations across modeling strategies, whether evaluated per standard deviation increase or according to the optimal cutoff value (adjusted HR per standard deviation increase: 1.213, 95% CI 1.048–1.403; adjusted HR for high vs. low SII: 1.717, 95% CI 1.109–2.656; both *p* < 0.05). RCS analysis revealed a significant linear positive association between SII and mortality risk (*P* for overall = 0.032; *P* for nonlinearity = 0.305). Subgroup analyses indicated significant associations between higher SII and all-cause mortality among patients aged ≥60 years, females, and those without anti-synthetase syndrome.

**Conclusion:**

Elevated SII is independently and linearly associated with increased all-cause mortality in patients with ILD. These findings suggest that SII may have potential clinical value in the assessment and management of patients with ILD.

## 1 Introduction

Interstitial lung disease (ILD) represents a heterogeneous group of pulmonary disorders characterized by chronic inflammation and fibrosis of the lung interstitium, which can ultimately lead to irreversible respiratory failure and high mortality (1). ILD comprises multiple subtypes, including idiopathic pulmonary fibrosis (IPF), connective tissue disease–associated ILD, and exposure-related ILD (1). Although the rate of disease progression varies across subtypes, the overall prognosis for most patients remains poor (1). IPF, in particular, is associated with an especially unfavorable outcome, with a median survival of approximately 2–5 years after diagnosis and a 5-year survival rate of less than 50%, underscoring the severe and progressive nature of the disease (2–4). The pathophysiology of ILD involves immune dysregulation and persistent chronic inflammation, processes that not only drive the progression of pulmonary fibrosis but may also contribute to systemic immune disturbance and multi-organ involvement (1). Consequently, early identification of high-risk patients and timely therapeutic intervention are of substantial clinical importance for improving outcomes in this population.

Chronic inflammation is closely associated with prognosis in patients with ILD (5). Conventional inflammatory markers, such as C-reactive protein (CRP) and erythrocyte sedimentation rate (ESR), are commonly used to assess disease activity; however, single biomarkers often lack sufficient sensitivity and specificity (6). Consequently, recent studies have focused on composite hematological indices that better capture the overall immune–inflammatory status, with the aim of improving prediction of disease progression and mortality risk (7–9). The systemic immune-inflammation index (SII) is one such marker, calculated as platelet count × neutrophil count/lymphocyte count (10). This index reflects both inflammatory burden and immune competence, integrating pro-inflammatory and immune components into a single measure, and has shown potential prognostic value across a range of diseases (11–13). SII was initially introduced in oncological research as a prognostic indicator, where elevated values were consistently associated with poorer survival outcomes, highlighting the role of systemic inflammation in disease progression and mortality (10). More recently, the prognostic significance of SII has been explored in non-malignant conditions, including cardiovascular and infectious diseases (14–16). For instance, higher admission SII has been identified as an independent predictor of in-hospital mortality in patients with infective endocarditis (17). In addition, studies in patients with chronic obstructive pulmonary disease (COPD) have demonstrated a positive association between elevated SII and all-cause mortality, further underscoring the relevance of inflammation-based indices in chronic lung diseases (18, 19). In the context of ILD, emerging evidence has begun to link inflammatory markers with disease severity and clinical outcomes. Several studies suggest that indices such as the neutrophil-to-lymphocyte ratio (NLR) are associated with disease progression, and higher NLR values have been inversely correlated with overall survival in patients with IPF (20, 21). Moreover, preliminary data indicate that SII may serve as a potential prognostic marker in IPF, with higher SII values associated with worse survival outcomes, suggesting its utility in mortality risk assessment among ILD patients (22). However, most previous investigations were limited by relatively small sample sizes, focused on specific ILD subtypes, or primarily examined disease activity and progression rather than systematically evaluating the independent association between SII and all-cause mortality. In addition, comprehensive multivariable adjustment and dose–response analyses have seldom been performed in this context. Therefore, the incremental prognostic value of SII for predicting all-cause mortality across a broader ILD population remains insufficiently clarified.

Therefore, investigating the prognostic value of SII in patients with ILD is of both theoretical and clinical importance. As a readily available and low-cost index derived from routine blood tests, SII may offer a practical tool for risk stratification and individualized management if its association with all-cause mortality is confirmed. In the present cohort study, we aimed to comprehensively evaluate the relationship between SII and all-cause mortality in patients with ILD, to determine whether SII serves as an independent prognostic marker, and to explore its performance across clinically relevant subgroups, thereby providing evidence to inform prognostic modeling and risk assessment strategies in this population.

## 2 Methods

### 2.1 Study population

This retrospective cohort study was conducted at The Second Affiliated Hospital of Soochow University. Consecutive patients diagnosed with ILD between March 2007 and February 2020 were screened for inclusion. ILD was diagnosed based on clinical manifestations, serological findings, and high-resolution computed tomography (HRCT), with or without histopathological confirmation, according to established criteria (23).

Inclusion criteria: (1) Age ≥ 18 years; (2) Diagnosis of ILD based on clinical, serological, and HRCT findings (with or without histopathological confirmation); (3) Availability of complete baseline data at admission, defined as: Demographic variables (age, sex, smoking status); Disease-related characteristics (anti-synthetase syndrome [ASS] status, anti–melanoma differentiation–associated gene 5 [anti-MDA5] antibody status, ILD type, and treatment information); Other key laboratory indices included in the multivariable analyses. Exclusion criteria: (1) Missing key laboratory variables required for calculation of the SII (platelet count, neutrophil count, and lymphocyte count); (2) Incomplete follow-up information, including unknown survival status or unavailable follow-up duration. A total of 366 patients were included in the final analysis.

This study adhered to the ethical standards outlined in the Declaration of Helsinki and was approved by the Ethics Committee of The Second Affiliated Hospital of Soochow University (JD-HG-2025-143). Given the retrospective design, the requirement for written informed consent was waived by the institutional ethics committee.

### 2.2 Data collection and variable definitions

Baseline demographic, clinical, and laboratory data were obtained from electronic medical records at the time of initial hospitalization. Demographic variables included age, sex, and smoking status (categorized as smoker or non-smoker). Clinical variables included ASS (yes/no), anti-MDA5 antibody positivity (yes/no), ILD type, combination therapy type, and disease duration.

In the present study, ILD type was categorized as acute or chronic based on the mode of clinical onset and the rate of disease progression, rather than on standard etiological classifications recommended by international guidelines. Acute ILD was defined as acute or subacute onset with rapid respiratory deterioration, whereas chronic ILD referred to a more indolent disease course (23). We acknowledge that this classification reflects clinical course characteristics and does not correspond to formal etiological subtypes of ILD. Combination therapy type was categorized into three groups: no treatment, glucocorticoids alone, and glucocorticoids plus immunosuppressants.

Laboratory variables included white blood cell count (WBC), neutrophil count, lymphocyte count, platelet count, hemoglobin, albumin, glucose, creatinine, C-reactive protein (CRP), erythrocyte sedimentation rate (ESR), complement C3, complement C4, immunoglobulin A (IgA), immunoglobulin E (IgE), immunoglobulin M (IgM), and immunoglobulin G (IgG). All laboratory measurements were performed using standard methods in the hospital’s certified clinical laboratory.

### 2.3 Definition and categorization of the systemic immune-inflammation index

The SII was calculated using the following formula: SII = platelet count (x10^9^/L) × neutrophil count (x10^9^/L) / lymphocyte count (x10^9^/L) (10). SII was analyzed as both a continuous variable and a categorical variable. The optimal cutoff value for SII was determined using receiver operating characteristic (ROC) curve analysis based on all-cause mortality, and the cutoff point was identified by maximizing the Youden index (sensitivity + specificity − 1). Patients were subsequently classified into low SII (≤ 1614.86) and high SII (> 1614.86) groups. In addition, SII was standardized using a *z*-score transformation, calculated as (SII − mean SII) divided by the standard deviation (SD) of SII, and analyzed per one SD increase in regression models.

### 2.4 Follow-up and outcome assessment

The primary outcome was all-cause mortality. Patients were followed from the date of ILD diagnosis until death or April 2020, whichever occurred first. Survival status was ascertained through hospital medical records and telephone follow-up when necessary. The median follow-up duration was 20.6 months. Patients who were alive at the end of follow-up were censored.

### 2.5 Statistical analysis

Continuous variables were first assessed for normality using the Shapiro–Wilk test. Variables with a normal distribution are presented as mean ± SD, whereas non-normally distributed variables were expressed as median (interquartile range). Comparisons between groups were performed using the Student’s t test for normally distributed variables and the Mann–Whitney *U* test for non-normally distributed variables. Categorical variables were presented as numbers (percentages) and were compared using the chi-square test or Fisher’s exact test, as appropriate.

The discriminatory ability of the SII for predicting all-cause mortality was evaluated using ROC curve analysis. Survival curves were generated using the Kaplan–Meier method and compared using the log-rank test. Univariable and multivariable Cox proportional hazards regression models were used to estimate hazard ratios (HRs) and 95% confidence intervals (CIs) for the association between SII and all-cause mortality. Variables with *p* < 0.05 in univariable Cox regression analyses were entered into the multivariable model, with additional consideration of clinical relevance, to reduce the risk of overfitting and ensure model interpretability. A stepwise adjustment strategy was applied to construct progressively adjusted models in order to evaluate the stability of the association across different levels of covariate adjustment. The proportional hazards assumption was formally tested using Schoenfeld residuals, and no significant violations were detected. Restricted cubic spline (RCS) analysis was applied to explore the dose–response relationship between SII and mortality risk. Prespecified subgroup analyses were conducted to assess the robustness and consistency of the association across clinically relevant subgroups. Patients with missing key laboratory variables required for SII calculation or incomplete follow-up information were excluded at baseline. No imputation methods were applied for missing data; therefore, all analyses were conducted using complete-case data.

All statistical analyses were performed using SPSS software (version 28.0; IBM Corp., Armonk, NY, United States) and R software (version 4.4.3; R Foundation for Statistical Computing, Vienna, Austria). A two-sided *p*-value < 0.05 was considered statistically significant.

## 3 Results

### 3.1 Baseline characteristics of the study population by mortality status

Table 1 summarized the baseline characteristics of the study population stratified by all-cause mortality. A total of 366 patients with ILD were included, among whom 91 deaths (24.9%) occurred during a median follow-up period of 20.6 months. Patients who died were significantly older than those who survived (61.13 ± 10.44 vs. 53.94 ± 11.15 years, *p* < 0.001). No significant differences were observed between the two groups with respect to sex distribution or smoking status (both *p* > 0.05).

**Table 1:** Baseline characteristics of the study population by mortality status.

Variables	Overall	Non-all-cause mortality	All-cause mortality	*P* value
*N*	366	275	91	
Age, years	55.73 ± 11.40	53.94 ± 11.15	61.13 ± 10.44	<0.001
Sex, *n* (%)				0.415
Male	220 (60.11%)	162 (58.91%)	58 (63.74%)	
Female	146 (39.89%)	113 (41.09%)	33 (36.26%)	
Smoking, *n* (%)	73 (19.95%)	57 (20.73%)	16 (17.58%)	0.515
Anti-synthetase syndrome, *n* (%)				<0.001
No	207 (56.56%)	138 (50.18%)	69 (75.82%)	
Yes	159 (43.44%)	137 (49.82%)	22 (24.18%)	
Anti-MDA5 positive, *n* (%)				<0.001
No	296 (80.87%)	247 (89.82%)	49 (53.85%)	
Yes	70 (19.13%)	28 (10.18%)	42 (46.15%)	
ILD type, *n* (%)				0.102
Chronic	295 (80.60%)	227 (82.55%)	68 (74.73%)	
Acute	71 (19.40%)	48 (17.45%)	23 (25.27%)	
Combination therapy type, *n* (%)				0.032
No treatment	48 (13.11%)	29 (10.55%)	19 (20.88%)	
Glucocorticoids alone	214 (58.47%)	168 (61.09%)	46 (50.55%)	
Glucocorticoids plus immunosuppressants	104 (28.42%)	78 (28.36%)	26 (28.57%)	
Disease duration, months	2.00 (1.00, 6.00)	2.00 (1.00, 6.00)	2.00 (1.00, 6.00)	0.648
White blood cell count, x10^9^/L	7.40 (5.60, 9.60)	7.30 (5.60, 9.60)	7.50 (5.80, 10.20)	0.700
Neutrophil count, x10^9^/L	5.55 (3.80, 7.70)	5.50 (3.60, 7.50)	6.00 (4.10, 8.80)	0.097
Lymphocyte count, x10^9^/L	1.20 (0.80, 1.60)	1.20 (0.90, 1.70)	0.80 (0.50, 1.20)	<0.001
Hemoglobin, g/L	128.32 ± 16.14	129.91 ± 15.33	123.53 ± 17.58	0.002
Platelet count, x10^9^/L	219.50 (181.00, 268.00)	220.00 (186.00, 271.00)	217.00 (165.00, 260.00)	0.119
Complement C3, g/L	1.12 (1.02, 1.23)	1.12 (1.04, 1.24)	1.09 (0.94, 1.19)	0.015
Complement C4, g/L	0.25 (0.20, 0.27)	0.24 (0.19, 0.27)	0.25 (0.22, 0.30)	0.009
Immunoglobulin A, g/L	2.64 (1.88, 3.22)	2.54 (1.81, 3.05)	2.73 (2.18, 3.80)	0.003
Immunoglobulin E, IU/mL	103.00 (49.00, 214.00)	90.00 (44.00, 196.00)	139.00 (70.00, 228.00)	0.001
Immunoglobulin M, g/L	1.50 (0.97, 1.92)	1.51 (1.02, 1.95)	1.49 (0.87, 1.84)	0.253
Immunoglobulin G, g/L	12.10 (10.80, 14.70)	12.00 (10.60, 14.70)	12.30 (11.00, 15.20)	0.425
Albumin, g/L	34.80 (31.80, 37.80)	36.00 (33.00, 38.60)	31.80 (29.10, 34.10)	<0.001
Glucose, mmol/L	5.12 (4.48, 6.70)	4.94 (4.44, 6.25)	6.17 (4.85, 8.14)	<0.001
Creatinine, μmol/L	50.00 (42.40, 60.00)	50.00 (43.00, 60.00)	50.00 (41.00, 61.00)	0.378
C-reactive protein, mg/L	7.00 (4.10, 20.90)	6.00 (3.80, 15.90)	19.80 (6.40, 50.70)	<0.001
Erythrocyte sedimentation rate, mm/h	29.00 (16.00, 44.00)	26.00 (13.00, 40.00)	38.00 (23.00, 60.00)	<0.001
SII	1,096.16 (574.41, 1,933.13)	1,017.21 (522.77, 1,725.50)	1,471.14 (892.40, 3,264.00)	<0.001
Standardized SII	−0.29 (−0.58, 0.17)	−0.34 (−0.61, 0.06)	−0.08 (−0.40, 0.92)	<0.001
Categorical variable				<0.001
Low SII (≤1614.86)	251 (68.58%)	203 (73.82%)	48 (52.75%)	
High SII (>1614.86)	115 (31.42%)	72 (26.18%)	43 (47.25%)	

SII, systemic immune-inflammation index; MDA5, melanoma differentiation-associated gene 5; ILD, interstitial lung disease.

Regarding disease-related characteristics, the prevalence of ASS was significantly lower in the mortality group compared with survivors (24.18% vs. 49.82%, *p* < 0.001), whereas anti-MDA5 antibody positivity was markedly more frequent among patients who died (46.15% vs. 10.18%, *p* < 0.001). The distribution of ILD type (acute vs. chronic) did not differ significantly between the two groups. In terms of treatment, a higher proportion of patients in the mortality group received no treatment, while glucocorticoid monotherapy was less common, and the overall difference in treatment patterns was statistically significant (*p* = 0.032).

Laboratory findings showed that lymphocyte counts, hemoglobin levels, and serum albumin concentrations were significantly lower in patients who died than in survivors (all *p* < 0.01), whereas neutrophil counts tended to be higher. With respect to immunological parameters, patients in the mortality group had lower complement C3 levels but higher complement C4, IgA, and IgE levels (all *p* < 0.05). No significant differences were observed in IgM or IgG levels between groups.

Among metabolic and inflammatory markers, glucose levels were significantly elevated in the mortality group, while serum creatinine levels were comparable between groups. Inflammatory markers, including CRP and ESR, were substantially higher in patients who died (both *p* < 0.001). Notably, both the SII and standardized SII values were significantly increased in the mortality group (both *p* < 0.001). When patients were classified according to the optimal SII cutoff value, the proportion of all-cause mortality was significantly higher in the high SII group than in the low SII group (47.25% vs. 26.18%, *p* < 0.001).

### 3.2 Baseline characteristics stratified by the optimal cutoff value of SII

Table 2 presented the baseline characteristics of patients stratified according to the optimal cutoff value of the SII. Among the 366 patients included, 251 were classified into the low SII group and 115 into the high SII group.

**Table 2:** Baseline characteristics stratified by the optimal cutoff value of SII.

Variables	Low SII	High SII	*P* value
*N*	251	115	
Age, years	55.48 ± 10.78	56.26 ± 12.68	0.569
Sex, *n* (%)			0.666
Male	149 (59.36%)	71 (61.74%)	
Female	102 (40.64%)	44 (38.26%)	
Smoking, n (%)	51 (20.32%)	22 (19.13%)	0.792
Anti-synthetase syndrome, *n* (%)			0.071
No	134 (53.39%)	73 (63.48%)	
Yes	117 (46.61%)	42 (36.52%)	
Anti-MDA5 positive, *n* (%)			0.045
No	210 (83.67%)	86 (74.78%)	
Yes	41 (16.33%)	29 (25.22%)	
ILD type, *n* (%)			0.182
Chronic	207 (82.47%)	88 (76.52%)	
Acute	44 (17.53%)	27 (23.48%)	
Combination therapy type, *n* (%)			0.436
No treatment	30 (11.95%)	18 (15.65%)	
Glucocorticoids alone	152 (60.56%)	62 (53.91%)	
Glucocorticoids plus immunosuppressants	69 (27.49%)	35 (30.43%)	
Disease duration, months	3.00 (1.00, 9.00)	2.00 (1.00, 5.00)	0.036
White blood cell count, x10^9^/L	6.70 (5.10, 8.30)	9.90 (7.40, 13.20)	<0.001
Neutrophil count, x10^9^/L	4.50 (3.20, 6.10)	8.60 (6.40, 11.50)	<0.001
Lymphocyte count, x10^9^/L	1.40 (1.00, 1.80)	0.80 (0.50, 1.10)	<0.001
Hemoglobin, g/L	129.76 ± 15.44	125.17 ± 17.22	0.015
Platelet count, x10^9^/L	206.00 (171.00, 247.00)	257.00 (221.00, 312.00)	<0.001
Complement C3, g/L	1.12 (1.03, 1.24)	1.12 (1.00, 1.22)	0.454
Complement C4, g/L	0.25 (0.20, 0.27)	0.24 (0.19, 0.28)	0.379
Immunoglobulin A, g/L	2.62 (1.88, 3.08)	2.73 (1.87, 3.66)	0.204
Immunoglobulin E, IU/mL	97.00 (46.00, 214.00)	122.00 (61.00, 214.00)	0.109
Immunoglobulin M, g/L	1.42 (0.94, 1.81)	1.64 (1.12, 2.23)	0.026
Immunoglobulin G, g/L	12.10 (10.80, 14.40)	12.10 (10.80, 15.20)	0.825
Albumin, g/L	35.60 (32.60, 38.40)	33.00 (30.20, 36.40)	<0.001
Glucose, mmol/L	4.82 (4.32, 6.06)	6.25 (5.03, 8.08)	<0.001
Creatinine, μmol/L	52.00 (44.00, 62.00)	47.00 (41.00, 58.00)	0.003
C-reactive protein, mg/L	5.80 (3.70, 15.90)	15.50 (4.90, 46.70)	<0.001
Erythrocyte sedimentation rate, mm/h	25.00 (13.00, 38.00)	38.00 (23.00, 56.00)	<0.001
All-cause mortality, *n* (%)			<0.001
No	203 (80.88%)	72 (62.61%)	
Yes	48 (19.12%)	43 (37.39%)	

SII, systemic immune-inflammation index; MDA5, melanoma differentiation-associated gene 5; ILD, interstitial lung disease.

There were no significant differences between the two groups with respect to age, sex distribution, or smoking status (all *p* > 0.05). Regarding disease-related characteristics, anti-MDA5 antibody positivity was significantly more frequent in the high SII group than in the low SII group (25.22% vs. 16.33%, *p* = 0.045). In contrast, the prevalence of ASS tended to be lower in the high SII group, although this difference did not reach statistical significance. No significant differences were observed between groups in terms of ILD type (acute vs. chronic) or treatment regimens.

Comparisons of laboratory parameters showed that patients in the high SII group had significantly higher WBC, neutrophil counts, and platelet counts, accompanied by a marked reduction in lymphocyte counts (all *p* < 0.001). In addition, hemoglobin levels were modestly lower (*p* = 0.015), and serum albumin levels were substantially reduced (*p* < 0.001) in the high SII group. With respect to immunological indices, levels of complement C3, complement C4, IgA, and IgE were comparable between groups, whereas IgM levels were significantly higher in the high SII group (*p* = 0.026). Among metabolic markers, glucose levels were significantly elevated in patients with high SII, while serum creatinine levels were slightly lower, with the difference reaching statistical significance. Inflammatory markers, including CRP and ESR, were markedly higher in the high SII group than in the low SII group (both *p* < 0.001).

Importantly, the incidence of all-cause mortality differed markedly between the two groups. Patients in the high SII group experienced a significantly higher proportion of all-cause mortality compared with those in the low SII group (37.39% vs. 19.12%, *p* < 0.001).

### 3.3 Univariable associations between clinical factors and all-cause mortality

Table 3 summarized the results of univariable Cox regression analyses examining the associations between clinical and laboratory variables and the risk of all-cause mortality. Increasing age was significantly associated with a higher risk of mortality (HR = 1.045, 95% CI: 1.026–1.064, *p* < 0.001), whereas no significant associations were observed for sex or smoking status.

**Table 3:** Univariable associations with all-cause mortality assessed by Cox regression.

Variables	HR	95% CI	*P*-value
Age	1.045	1.026, 1.064	<0.001
Sex			
Male	1.142	0.744, 1.752	0.544
Female	—	—	
Smoking	0.916	0.533, 1.574	0.752
Anti-synthetase syndrome			
No	—	—	
Yes	0.373	0.231, 0.603	<0.001
Anti-MDA5 positive			
No	—	—	
Yes	6.998	4.537, 10.795	<0.001
ILD type			
Chronic	—	—	
Acute	1.636	1.017, 2.630	0.042
Combination therapy type			
No treatment	—	—	
Glucocorticoids alone	0.443	0.259, 0.756	0.003
Glucocorticoids plus immunosuppressants	0.509	0.281, 0.922	0.026
Disease duration	0.992	0.980, 1.004	0.177
White blood cell count	1.047	0.993, 1.103	0.088
Neutrophil count	1.083	1.030, 1.140	0.002
Lymphocyte count	0.280	0.182, 0.433	<0.001
Hemoglobin	0.980	0.968, 0.992	0.001
Platelet count	0.998	0.994, 1.001	0.135
Complement C3	0.175	0.050, 0.608	0.006
Complement C4	245.117	13.228, 4,542.161	<0.001
Immunoglobulin A	1.317	1.122, 1.547	<0.001
Immunoglobulin E	1.000	1.000, 1.001	0.149
Immunoglobulin M	0.904	0.714, 1.143	0.398
Immunoglobulin G	1.036	0.984, 1.091	0.181
Albumin	0.856	0.825, 0.888	<0.001
Glucose	1.257	1.172, 1.349	<0.001
Creatinine	0.992	0.976, 1.008	0.317
C-reactive protein	1.016	1.011, 1.021	<0.001
Erythrocyte sedimentation rate	1.020	1.012, 1.028	<0.001
SII	1.000	1.000, 1.000	<0.001
Standardized SII	1.385	1.259, 1.524	<0.001
Categorical variable			
Low SII (≤1614.86)	—	—	
High SII (>1614.86)	2.491	1.644, 3.775	<0.001

SII, systemic immune-inflammation index; MDA5, melanoma differentiation-associated gene 5; ILD, interstitial lung disease.

With respect to disease-related characteristics, the presence of ASS was associated with a significantly lower risk of death (HR = 0.373, 95% CI: 0.231–0.603, *p* < 0.001). In contrast, anti-MDA5 antibody positivity was strongly associated with an increased risk of all-cause mortality (HR = 6.998, 95% CI: 4.537–10.795, p < 0.001). Patients with acute ILD also had a higher mortality risk compared with those with chronic ILD (HR = 1.636, 95% CI: 1.017–2.630, *p* = 0.042).

Regarding treatment patterns, both glucocorticoid monotherapy and glucocorticoids combined with immunosuppressive agents were associated with a reduced risk of all-cause mortality compared with no treatment (HR = 0.443 and HR = 0.509, respectively; both *p* < 0.05). Among laboratory parameters, higher neutrophil counts were associated with an increased risk of death (HR = 1.083, *p* = 0.002), whereas higher lymphocyte counts, hemoglobin levels, and serum albumin concentrations were each significantly associated with a lower mortality risk (all *p* < 0.05). In immunological analyses, complement C3 levels were inversely associated with mortality risk (HR = 0.175, *p* = 0.006), while elevated complement C4 and IgA levels were associated with a significantly increased risk of death (both *p* < 0.001). Among metabolic and inflammatory markers, higher glucose levels, CRP, and ESR were all significantly associated with increased mortality risk (all *p* < 0.05), whereas serum creatinine showed no significant association.

Notably, SII was consistently associated with all-cause mortality across different analytical approaches. When analyzed as a standardized variable, each one–SD increase in SII was associated with a 38.5% increase in mortality risk (HR = 1.385, 95% CI: 1.259–1.524, *p* < 0.001). Similarly, patients in the high SII group had a significantly higher risk of death compared with those in the low SII group (HR = 2.491, 95% CI: 1.644–3.775, *p* < 0.001).

### 3.4 Adjusted associations between SII and all-cause mortality

The association between SII and all-cause mortality was further evaluated using multivariable Cox proportional hazards models, as shown in Table 4. SII was analyzed as both a continuous variable and a categorical variable based on the optimal cutoff value.

**Table 4:** Association between SII and the risk of all-cause mortality.

Variables	Model 1			Model 2			Model 3		
	HR	95% CI	*P*-value	HR	95% CI	*P*-value	HR	95% CI	*P*-value
Continuous variable									
SII	1.000	1.000, 1.000	<0.001	1.000	1.000, 1.000	<0.001	1.000	1.000, 1.000	0.009
Standardized SII	1.379	1.246, 1.527	<0.001	1.461	1.306, 1.635	<0.001	1.213	1.048, 1.403	0.009
Categorical variable									
Low SII	Ref			Ref			Ref		
High SII	2.449	1.613, 3.717	<0.001	2.191	1.428, 3.362	<0.001	1.717	1.109, 2.656	0.015

Model 1: adjusted for age only; Model 2: adjusted for age, anti-synthetase syndrome, anti-MDA5 positive, ILD type, and combination therapy type; Model 3: adjusted for age, anti-synthetase syndrome, anti-MDA5 positivity, ILD type, hemoglobin, complement C3, complement C4, immunoglobulin A, albumin, glucose, C-reactive protein, erythrocyte sedimentation rate, and combination therapy type. SII, systemic immune-inflammation index; MDA5, melanoma differentiation-associated gene 5; ILD, interstitial lung disease; HR, hazard ratio; CI, confidence interval.

In Model 1, which was adjusted for age only, higher SII was significantly associated with an increased risk of all-cause mortality. When SII was analyzed as a standardized variable, each one–SD increase in SII was associated with a 37.9% higher mortality risk (HR = 1.379, 95% CI: 1.246–1.527, *p* < 0.001). Similarly, patients in the high SII group had a significantly higher risk of death compared with those in the low SII group (HR = 2.449, 95% CI: 1.613–3.717, *p* < 0.001).

After further adjustment for disease-related characteristics and treatment factors in Model 2, including ASS, anti-MDA5 antibody positivity, ILD type, and combination therapy type, the association between SII and all-cause mortality remained robust. Standardized SII continued to show a strong association with mortality (HR = 1.461, 95% CI: 1.306–1.635, *p* < 0.001), and high SII was associated with more than a twofold increase in mortality risk (HR = 2.191, 95% CI: 1.428–3.362, *p* < 0.001).

In the fully adjusted Model 3, which additionally accounted for key laboratory parameters and inflammatory markers, SII remained an independent predictor of all-cause mortality. Each one–SD increase in standardized SII was associated with a 21.3% increase in mortality risk (HR = 1.213, 95% CI: 1.048–1.403, *p* = 0.009). Consistently, patients in the high SII group had a significantly higher risk of death compared with those in the low SII group (HR = 1.717, 95% CI: 1.109–2.656, *p* = 0.015).

### 3.5 Subgroup analyses of the association between SII and all-cause mortality

Adjusted subgroup analyses examining the association between SII and all-cause mortality were presented in Table 5. These subgroup analyses were prespecified during the study design phase and were conducted according to age (<60 vs. ≥60 years), sex (male vs. female), and ASS status (yes vs. no). Analyses were conducted using both categorical SII (high vs. low) and standardized SII, with adjustment for demographic characteristics, disease-related factors, laboratory indices, inflammatory markers, and treatment regimens.

**Table 5:** Adjusted subgroup analyses of SII and all-cause mortality.

Subgroups	SII (High vs. Low)			Standardized SII		
	HR	95% CI	*P*-value	HR	95% CI	*P*-value
Age						
<60 years	1.531	0.705, 3.328	0.282	1.003	0.816, 1.232	0.979
≥60 years	1.988	1.138, 3.474	0.016	1.521	1.254, 1.845	<0.001
Sex						
Male	1.137	0.646, 2.000	0.657	1.169	1.000, 1.367	0.051
Female	2.360	1.010, 5.518	0.047	1.821	1.394, 2.377	<0.001
Anti-synthetase syndrome						
Yes	1.163	0.317, 4.266	0.820	1.764	1.068, 2.914	0.027
No	1.688	1.031, 2.766	0.038	1.181	1.023, 1.362	0.023

Subgroup analysis adjusted for age, anti-synthetase syndrome, anti-MDA5 positivity, ILD type, hemoglobin, complement C3, complement C4, immunoglobulin A, albumin, glucose, C-reactive protein, erythrocyte sedimentation rate, and combination therapy type. SII, systemic immune-inflammation index; MDA5, melanoma differentiation-associated gene 5; ILD, interstitial lung disease; HR, hazard ratio; CI, confidence interval.

In age-stratified analyses, a significant association between high SII and increased mortality risk was observed among patients aged ≥ 60 years (HR = 1.988, 95% CI: 1.138–3.474, *p* = 0.016), whereas no significant association was detected in patients aged < 60 years. Similarly, standardized SII was significantly associated with mortality in the older age group (HR = 1.521, 95% CI: 1.254–1.845, *p* < 0.001), but not in younger patients.

When stratified by sex, high SII was associated with a significantly increased risk of all-cause mortality among female patients (HR = 2.360, 95% CI: 1.010–5.518, *p* = 0.047), whereas no significant association was observed in male patients. Consistent results were observed when SII was analyzed as a standardized variable, with a stronger association seen in females (HR = 1.821, 95% CI: 1.394–2.377, *p* < 0.001).

In subgroup analyses according to ASS status, high SII was significantly associated with increased mortality risk in patients without ASS (HR = 1.688, 95% CI: 1.031–2.766, *p* = 0.038), but not in those with ASS. However, when analyzed as a standardized variable, SII was significantly associated with mortality in both subgroups, with HRs of 1.764 (95% CI: 1.068–2.914, *p* = 0.027) in patients with ASS and 1.181 (95% CI: 1.023–1.362, *p* = 0.023) in those without.

### 3.6 Predictive performance, survival difference, and dose–response relationship of SII

**Figure 1:**
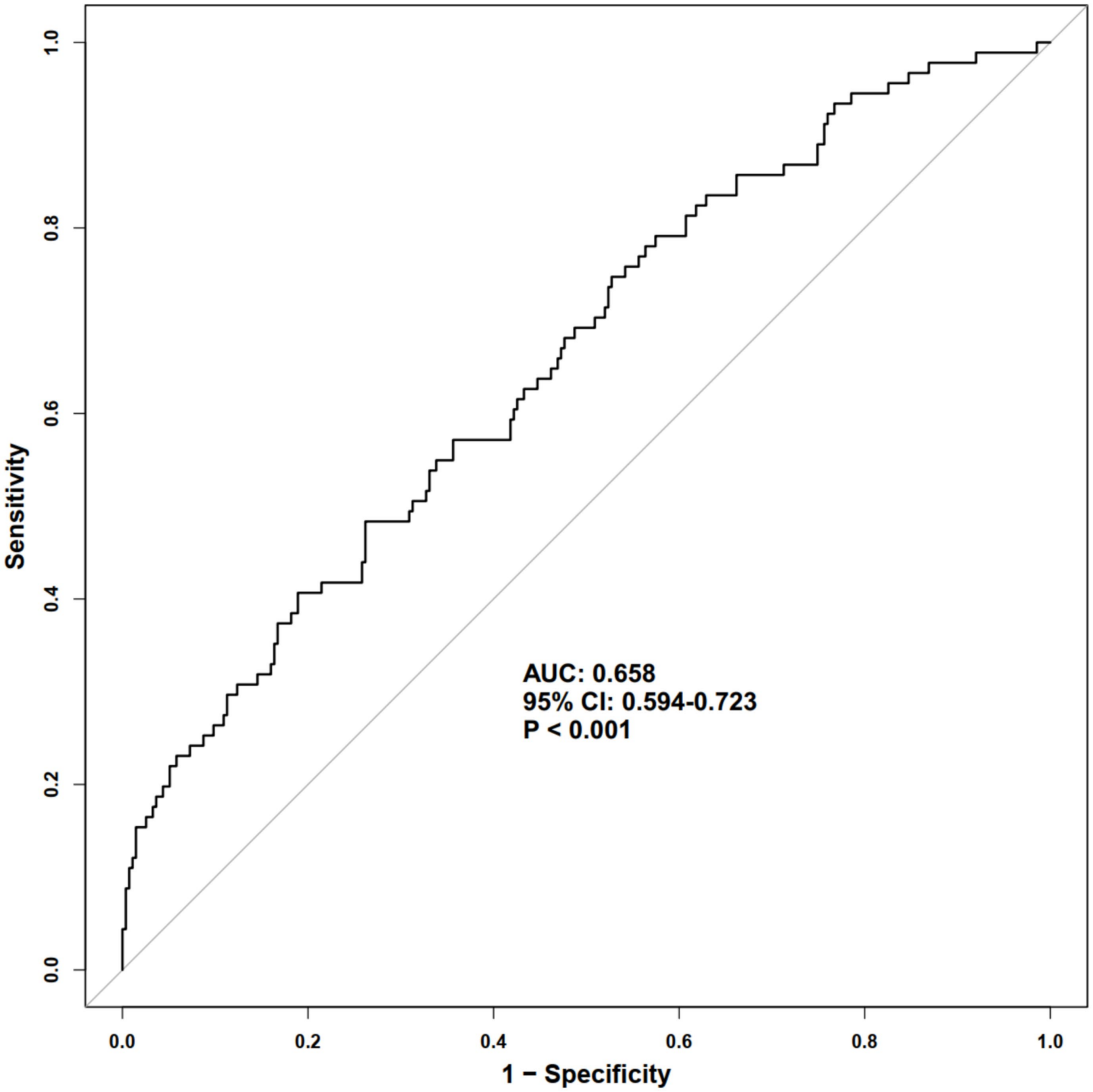
Predictive performance of the SII for all-cause mortality assessed by ROC analysis. SII, systemic immune-inflammation index; ROC, receiver operating characteristic; AUC, area under the curve; CI, confidence interval.

**Figure 2:**
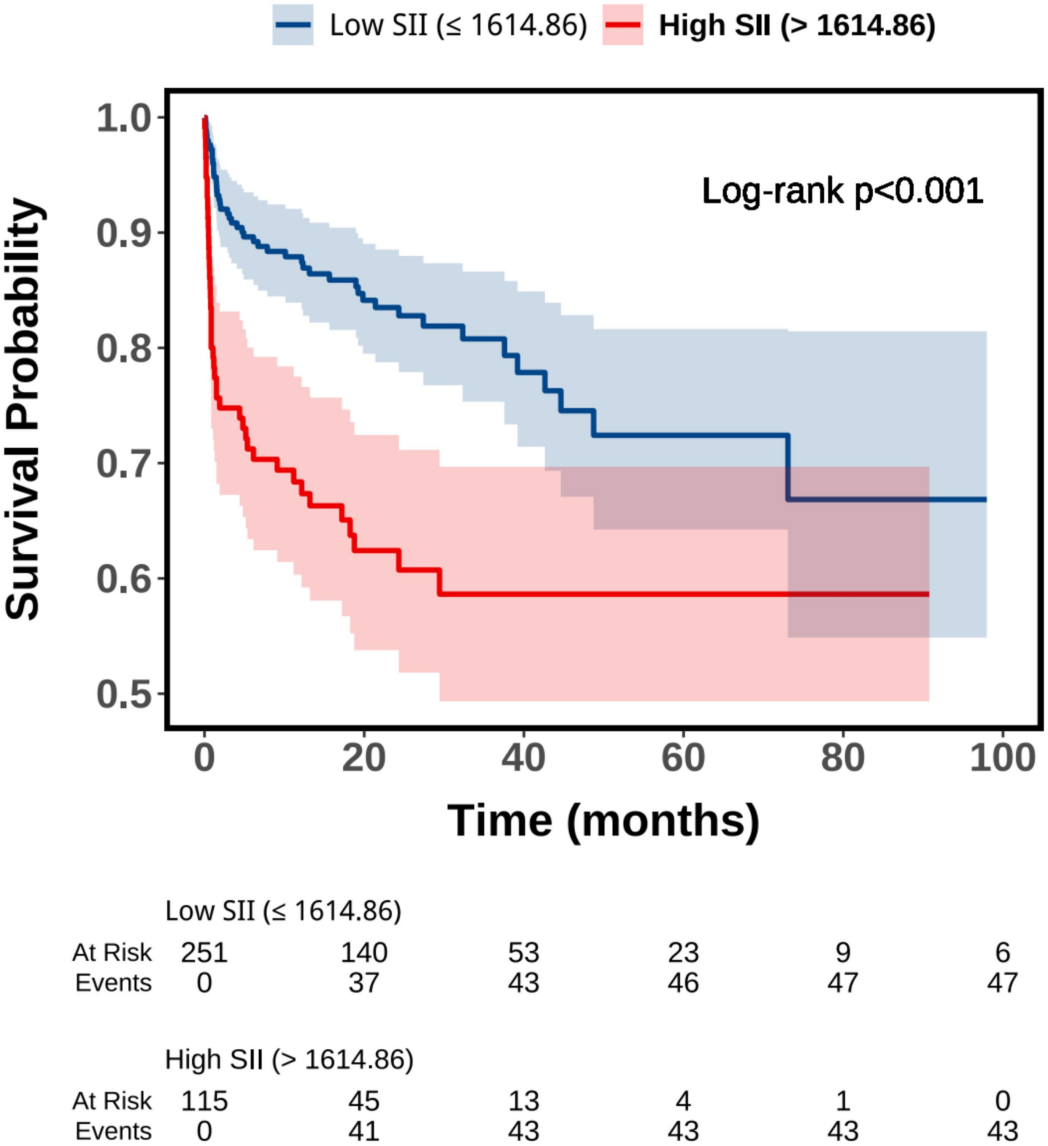
Kaplan–Meier survival analysis of all-cause mortality by SII categories. SII, systemic immune-inflammation index.

**Figure 3:**
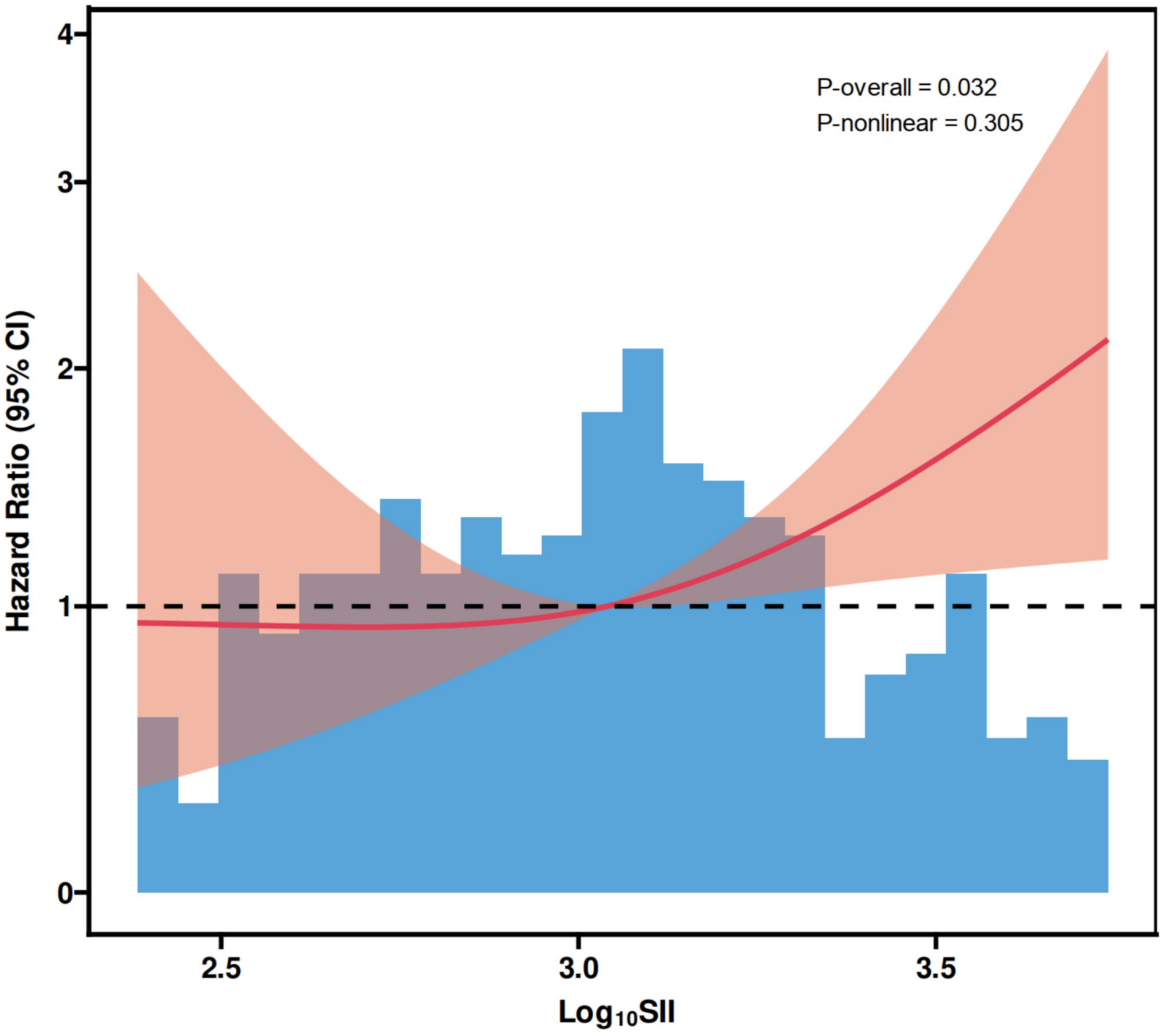
Dose–response relationship between SII and all-cause mortality evaluated using RCS. SII, systemic immune-inflammation index; RCS, restricted cubic spline; CI, confidence interval.

As shown in Figure 1, ROC curve analysis demonstrated that SII had a statistically significant ability to discriminate all-cause mortality, with an area under the curve (AUC) of 0.658 (95% CI: 0.594–0.723, *p* < 0.001). Based on the optimal cutoff value derived from the ROC analysis, patients were classified into low and high SII groups. Kaplan–Meier survival analysis revealed a significantly lower cumulative survival probability in patients with high SII compared with those with low SII (Figure 2), and the difference between the two survival curves was statistically significant (log-rank *p* < 0.001). Furthermore, RCS analysis showed a significant dose–response relationship between SII and the risk of all-cause mortality (Figure 3). The overall association was statistically significant (*P* for overall = 0.032), while no evidence of a nonlinear relationship was observed (*P* for non-linearity = 0.305), indicating a linear positive association between increasing SII levels and mortality risk.

## 4 Discussion

This study, based on a retrospective cohort, systematically examined the association between SII and the risk of all-cause mortality in patients with ILD. The findings showed that SII was significantly associated with mortality risk whether analyzed as a continuous variable, a standardized variable, or categorized according to the optimal cutoff value, and this association remained robust after adjustment for multiple confounding factors. Multivariable models were constructed using a stepwise adjustment strategy, and the proportional hazards assumption was formally tested, which supports the stability of the observed associations. Consistent results from survival analyses and dose–response modeling further demonstrated a linear positive relationship between increasing SII levels and mortality risk. However, given the relatively limited number of observations at the higher range of SII values, the uncertainty in this range should be interpreted with caution. Taken together, these results suggest that SII, a composite inflammation–immune marker derived from routine blood counts, reflects the overall disease status of patients with ILD and may have potential clinical relevance in patient assessment, while its incremental prognostic value beyond established clinical factors warrants further investigation.

In recent years, as composite hematological inflammatory indices have demonstrated potential value across a range of chronic diseases, the SII has gradually been introduced into research on ILD. However, the existing body of evidence remains heterogeneous, with substantial variability in study populations, research objectives, and outcome measures. As a result, the true prognostic significance of SII in ILD has not yet been clearly established. Early investigations of SII in ILD primarily focused on disease identification and the assessment of inflammatory status rather than mortality outcomes. The study by Ruta et al. (24) was among the first to systematically compare SII levels in patients with IPF and connective tissue disease–associated ILD (CTD-ILD). Their findings showed that SII values were elevated in both ILD groups compared with healthy controls; however, in the absence of acute exacerbation, SII was not significantly associated with prognosis. These results suggest that, in relatively stable ILD, SII may primarily reflect the presence of pulmonary involvement rather than serve as a direct indicator of mortality risk. Nevertheless, the interpretation of these findings was limited by the small sample size and the restricted scope of follow-up outcomes. Subsequently, attention shifted toward the discriminatory value of SII among different ILD subtypes. Sargin et al. (25) reported significant differences in SII levels among patients with Sjögren’s syndrome–associated ILD, interstitial pneumonia with autoimmune features (IPAF), and IPF, and observed correlations between SII and CRP in selected subgroups. These findings imply that SII may carry disease-specific phenotypic information. However, as the primary aim of that study was diagnostic differentiation, the relationship between SII and long-term survival or mortality was not examined, thereby limiting its prognostic implications. In the context of CTD-ILD, Xing et al. conducted a comprehensive comparison of multiple inflammatory and composite indices, including Krebs von den Lungen-6 (KL-6) and SII, for disease diagnosis and severity assessment (26). Although SII was found to correlate with impaired diffusing capacity, its independent predictive value was substantially weaker than that of KL-6 in multivariable analyses. This study highlighted the potential role of SII as a marker of inflammatory burden, while also suggesting limited stability when used alone as a prognostic indicator, particularly following therapeutic intervention. Studies focusing on specific autoimmune disease populations have provided additional insights. Ecesoy and Ecesoy (27) demonstrated that SII levels were significantly higher in patients with systemic sclerosis–associated ILD compared with healthy controls and were partially reflective of disease activity. While these findings further support the validity of SII as an indicator of inflammatory intensity, the predominantly cross-sectional design precluded conclusions regarding its role in predicting mortality. In ASS and anti-MDA5–related diseases, research on SII has increasingly emphasized the prediction of severe outcomes. Huang et al. (28) observed markedly elevated SII levels in ASS patients, with significant associations with ILD occurrence, fever, and infection, suggesting a role for SII in the systemic amplification of disease activity. Building on this, Yu et al. (29) demonstrated that SII was significantly higher in patients with rapidly progressive ILD (RPILD) and in non-survivors among anti-MDA5 dermatomyositis patients, and that SII contributed meaningfully to multivariable predictive models. Collectively, these findings indicate that SII may be particularly informative in ILD subgroups characterized by uncontrolled inflammation and extremely poor prognosis. Additionally, Tan et al. (30) examined the association between inflammatory markers and disease progression in a broader ILD population and identified SII as an independent predictor of ILD progression. However, their analysis focused primarily on disease progression rather than mortality, and the relatively short follow-up period limited the assessment of long-term outcomes. Taken together, existing studies on SII in ILD have largely concentrated on disease identification, inflammatory status evaluation, or severe outcomes in specific subtypes, with generally small sample sizes and inconsistent outcome definitions. Few investigations have systematically assessed the independent association between SII and all-cause mortality. Against this background, the present study leverages a larger real-world cohort encompassing multiple ILD subtypes to comprehensively evaluate the relationship between SII and all-cause mortality. Through stepwise multivariable adjustment, prespecified subgroup analyses, and dose–response modeling, our findings provide robust evidence that extends the current understanding of SII as a prognostic marker in ILD and broaden its potential application in clinical risk stratification. From a clinical perspective, these findings suggest that SII could be incorporated into routine assessment to facilitate early risk stratification, identify high-risk patients who may benefit from closer monitoring, and assist clinicians in tailoring follow-up intensity and therapeutic decision-making. Given its low cost and wide availability, SII may serve as a practical adjunct to existing clinical and functional indicators in daily practice. However, the cut-off value derived from ROC analysis was determined statistically within this cohort using the maximum Youden index and has not been externally validated; therefore, its clinical applicability should be interpreted with caution. Moreover, analyses treating SII as a continuous variable and exploring its dose–response relationship may provide a more comprehensive understanding of its association with mortality risk than reliance on a single fixed threshold.

In the course of ILD, an increased risk of death is often not determined solely by the extent of structural lung damage, but rather reflects the cumulative impact of multiple systemic pathological alterations over time. The SII, which integrates neutrophils, platelets, and lymphocytes, may therefore represent a composite peripheral blood signature of this broader systemic pathological state. From a holistic perspective, patients with ILD frequently exhibit a coexistence of persistent inflammatory activation and impaired immune regulation. On the one hand, sustained pro-inflammatory activity can exacerbate interstitial lung injury through multiple pathways and increase susceptibility to acute exacerbations or secondary infections (31). On the other hand, diminished immune regulatory capacity limits effective control of inflammatory responses, allowing inflammation to evolve into a systemic burden (32). An elevated SII is thus unlikely to reflect an abnormality of a single inflammatory pathway; instead, it may indicate an unstable state characterized by ongoing inflammation coupled with progressive exhaustion of immune compensatory mechanisms—a condition that is particularly detrimental in patients with ILD. Biologically, neutrophilia may reflect excessive innate immune activation and release of pro-fibrotic mediators, elevated platelet counts may contribute to microvascular injury and fibroblast activation through platelet-derived growth factors, whereas lymphopenia may indicate impaired adaptive immune regulation and immune exhaustion. The combined effect of these alterations, as captured by SII, may therefore promote a pro-inflammatory and pro-fibrotic milieu that accelerates disease progression and increases vulnerability to fatal complications. When considered alongside the clinical features observed in patients with high SII in this study, including poorer nutritional status, metabolic disturbances, and heightened inflammatory burden, SII appears to be closely linked to reduced overall physiological resilience. In this context, even when pulmonary disease progression itself is relatively slow, patients may still face an increased risk of death due to infections, metabolic imbalance, or failure of multiple organ systems. Accordingly, the risk captured by SII may primarily reflect amplification of systemic disease effects rather than the severity of pulmonary involvement alone. Moreover, heterogeneity in immune background across ILD subtypes may further influence the biological implications of SII. In disease contexts dominated by autoimmune dysregulation, a certain degree of inflammatory activation may constitute an intrinsic component of the disease process and may not uniformly translate into adverse outcomes (33). In contrast, among ILD patients without a clearly defined immune-driven mechanism, systemic inflammatory imbalance is more likely to represent a nonspecific but highly hazardous pathological condition. This context-dependent interpretation may partly explain the observed variability in the strength of association between SII and mortality across different subgroups. Finally, the linear relationship observed between SII and mortality risk suggests that SII does not merely capture episodic inflammatory events, but rather reflects a progressively accumulating state of systemic dysregulation over the disease course. As this imbalance intensifies, patients’ tolerance to acute stressors and complications diminishes, leading to a steady increase in mortality risk. Taken together, the prognostic significance of SII in patients with ILD may lie in its ability to characterize a composite pathological state marked by persistent systemic inflammation, impaired immune regulation, and depletion of overall physiological reserve—a constellation of factors that collectively underpins the elevated risk of all-cause mortality in this population.

This study has several limitations that should be considered when interpreting the findings. First, as a single-center retrospective cohort study, although the sample size was relatively large, the results may still be subject to selection bias and information bias, which could limit the generalizability of the conclusions. Second, SII was calculated solely based on baseline hematological data, and dynamic changes in SII during follow-up and their prognostic impact were not analyzed; this may have led to an underestimation of its long-term prognostic value. Third, ILD is a highly heterogeneous group of disorders, with substantial differences in underlying etiology, immuno-inflammatory mechanisms, and natural history across subtypes. In the present study, patients with diverse etiological backgrounds were largely analyzed together, and detailed subtype-specific classification was not consistently available due to the retrospective design and incomplete etiological data in medical records. Moreover, given the overall sample size, further stratification into major disease categories such as IPF, CTD-ILD, or exposure-related ILD would have resulted in relatively small subgroup sizes and insufficient statistical power. Therefore, the generalizability of our findings to specific ILD subtypes should be interpreted with caution. This heterogeneity may have introduced variability in baseline risk, inflammatory profiles, and treatment responses across patients, potentially attenuating or obscuring subtype-specific associations between SII and mortality. As a result, the observed overall association may not fully reflect differences in prognostic relevance across distinct ILD entities. Besides, although multivariable models and prespecified subgroup analyses were performed to adjust for several key factors, residual and unmeasured confounding cannot be completely excluded despite statistical adjustment. Fourth, lung function parameters, quantitative imaging features, and molecular or biomarker data were not included in the analysis. In particular, key indicators of disease severity such as pulmonary function parameters and quantitative HRCT data were not incorporated, which may introduce residual confounding and limit the ability to fully account for baseline disease severity. These data were not consistently available due to the retrospective design, incomplete documentation in earlier medical records, and the absence of standardized quantitative imaging assessments for all patients. This limitation restricts our ability to determine whether the observed association between SII and mortality is fully independent of disease severity and raises the possibility of residual confounding. If higher SII levels partly reflect more advanced functional impairment or more extensive fibrotic involvement, the strength of the observed association may, to some extent, be influenced by underlying disease severity. Fifth, although SII demonstrated statistically significant discriminatory ability, we were unable to formally evaluate its incremental prognostic value beyond established clinical characteristics. Specifically, we did not construct a standardized baseline clinical prediction model incorporating key prognostic variables such as lung function parameters or validated prognostic scores, nor did we perform formal model comparison analyses (e.g., C-statistic comparison, net reclassification improvement, or integrated discrimination improvement). In addition, the optimal cutoff value of SII (1614.86) was determined statistically within this single-center cohort using ROC analysis and has not been externally validated in independent populations. Therefore, its clinical applicability and generalizability should be interpreted with caution, and overinterpretation should be avoided. Finally, treatment strategies in real-world clinical practice often change over time, whereas this study only accounted for initial treatment patterns, which may not fully capture the long-term impact of therapeutic interventions on outcomes. Taken together, these limitations highlight the need for well-designed prospective studies to further validate the present findings.

## 5 Conclusion

In summary, this study is the first to systematically demonstrate, in a relatively large ILD cohort, an independent and linear positive association between SII and the risk of all-cause mortality. Owing to its simplicity, low cost, and wide availability, SII may serve as an adjunctive prognostic tool to complement existing clinical and imaging evaluations, providing additional information for patient assessment and risk stratification. However, it should not be used as a standalone indicator or replace established diagnostic and prognostic methods. The incremental predictive value of SII beyond conventional clinical factors remains to be further clarified. Therefore, future multicenter, prospective studies incorporating dynamic assessment of SII and analyses across ILD subtypes are warranted to validate its clinical utility and to explore how it may guide individualized treatment and follow-up strategies.

## Data Availability

The raw data supporting the conclusions of this article will be made available by the authors, without undue reservation.

## References

[1] Maher TM. Interstitial lung disease: a review. JAMA. (2024) 331:1655–65. doi: 10.1001/jama.2024.3669, 38648021

[2] Chen X Guo J Yu D Jie B Zhou Y. Predictors of mortality in progressive Fibrosing interstitial lung diseases. Front Pharmacol. (2021) 12:754851. doi: 10.3389/fphar.2021.754851, 34712141 PMC8546258

[3] Costabel U. Idiopathic pulmonary fibrosis: recent milestones in disease management. Eur Respir Rev. (2012) 21:140. doi: 10.1183/09059180.00000712, 22654085 PMC9487293

[4] Richeldi L Collard HR Jones MG. Idiopathic pulmonary fibrosis. Lancet. (2017) 389:1941–52. doi: 10.1016/S0140-6736(17)30866-8, 28365056

[5] Behr J Salisbury ML Walsh SLF Podolanczuk AJ Hariri LP Hunninghake GM . The role of inflammation and fibrosis in interstitial lung disease treatment decisions. Am J Respir Crit Care Med. (2024) 210:392–400. doi: 10.1164/rccm.202401-0048PP, 38484133 PMC12039538

[6] Harrison M. Erythrocyte sedimentation rate and C-reactive protein. Aust Prescr. (2015) 38:93–4. doi: 10.18773/austprescr.2015.034, 26648629 PMC4653962

[7] Liu K Tang S Liu C Ma J Cao X Yang X . Systemic immune-inflammatory biomarkers (SII, NLR, PLR and LMR) linked to non-alcoholic fatty liver disease risk. Front Immunol. (2024) 15:1337241. doi: 10.3389/fimmu.2024.1337241, 38481995 PMC10933001

[8] Liang Q Zhang S Wang X. Systemic immune-inflammatory biomarkers assist in differentiating clinical features of different etiologies of acute kidney injury: results from eICU collaborative research database. Ren Fail. (2025) 47:2525456. doi: 10.1080/0886022X.2025.2525456, 40603274 PMC12224723

[9] Fornarini G Rebuzzi SE Banna GL Calabrò F Scandurra G de Giorgi U . Immune-inflammatory biomarkers as prognostic factors for immunotherapy in pretreated advanced urinary tract cancer patients: an analysis of the Italian SAUL cohort. ESMO Open. (2021) 6:100118. doi: 10.1016/j.esmoop.2021.100118, 33984678 PMC8134706

[10] Hu B Yang XR Xu Y Sun Y-F Sun C Guo W . Systemic immune-inflammation index predicts prognosis of patients after curative resection for hepatocellular carcinoma. Clin Cancer Res. (2014) 20:6212–22. doi: 10.1158/1078-0432.CCR-14-0442, 25271081

[11] Ma F Li L Xu L Wu J Zhang A Liao J . The relationship between systemic inflammation index, systemic immune-inflammatory index, and inflammatory prognostic index and 90-day outcomes in acute ischemic stroke patients treated with intravenous thrombolysis. J Neuroinflammation. (2023) 20:220. doi: 10.1186/s12974-023-02890-y, 37777768 PMC10543872

[12] Huang P Mai Y Zhao J Yi Y Wen Y. Association of systemic immune-inflammation index and systemic inflammation response index with chronic kidney disease: observational study of 40,937 adults. Inflamm Res. (2024) 73:655–67. doi: 10.1007/s00011-024-01861-0, 38489048

[13] Zhang Y Han S Duan Z Tian X Li X Hou G . Associations of systemic inflammation and systemic immune inflammation with serum uric acid concentration and hyperuricemia risk: the mediating effect of body mass index. Front Endocrinol (Lausanne). (2024) 15:1469637. doi: 10.3389/fendo.2024.1469637, 39720251 PMC11667560

[14] Kurklu HA Tan TS. Systemic immune-inflammation index predicts post-MI left ventricular remodeling. Int J Cardiovasc Imaging. (2024) 40:991–1000. doi: 10.1007/s10554-024-03064-4, 38345664

[15] Li M Li M Wang Z Zhang Y. The combined effect of the systemic immune-inflammation index and aortic valve calcification on major adverse cardiovascular events in patients with coronary heart disease. J Inflamm Res. (2024) 17:8375–84. doi: 10.2147/JIR.S493735, 39529998 PMC11552382

[16] Kalyenci B Çoban F Sulhan H Yücel MÖ Benlioğlu C Kaz G . The role of Pan-immune inflammation value and systemic immune-inflammation index as potential biomarkers in predicting infectious complications following retrograde intrarenal surgery. BMC Urol. (2025) 25:168. doi: 10.1186/s12894-025-01859-8, 40660101 PMC12257762

[17] Agus HZ Kahraman S Arslan C Yildirim C Erturk M Kalkan AK . Systemic immune-inflammation index predicts mortality in infective endocarditis. J Saudi Heart Assoc. (2020) 32:58–64. doi: 10.37616/2212-5043.1010, 33154893 PMC7640593

[18] Ye C Yuan L Wu K Shen B Zhu C. Association between systemic immune-inflammation index and chronic obstructive pulmonary disease: a population-based study. BMC Pulm Med. (2023) 23:295. doi: 10.1186/s12890-023-02583-5, 37563621 PMC10416535

[19] Yi Q Chen X Liu S Luo Y Wei H Ge H . Systemic immune-inflammation index is associated with adverse outcomes in patients hospitalized for AECOPD: a multicenter cohort study. Respiration. (2025) 104:648–66. doi: 10.1159/000545267, 40233725

[20] Chen Y Cai J Zhang M Yan X. Prognostic role of NLR, PLR and MHR in patients with idiopathic pulmonary fibrosis. Front Immunol. (2022) 13:882217. doi: 10.3389/fimmu.2022.882217, 35572564 PMC9096781

[21] Mikolasch TA George PM Sahota J Nancarrow T Barratt SL Woodhead FA . Multi-center evaluation of baseline neutrophil-to-lymphocyte (NLR) ratio as an independent predictor of mortality and clinical risk stratifier in idiopathic pulmonary fibrosis. EClinicalMedicine. (2022) 55:101758. doi: 10.1016/j.eclinm.2022.101758 36483266 PMC9722446

[22] Koyun GB Berk S Dogan OT. The importance of SII and FIB-4 scores in predicting mortality in idiopathic pulmonary fibrosis patients. Clin Biochem. (2024) 131:110789. doi: 10.1016/j.clinbiochem.2024.110789 38977211

[23] Johnson SR Bernstein EJ Bolster MB Chung JH Danoff SK George MD . 2023 American College of Rheumatology (ACR)/American College of CHEST Physicians (CHEST) guideline for the screening and monitoring of interstitial lung disease in people with systemic autoimmune rheumatic diseases. Arthritis Rheum. (2024) 76:1201–13. doi: 10.1002/art.42860, 38973714 PMC12646464

[24] Ruta VM Man AM Alexescu TG Motoc NS Tarmure S Ungur RA . Neutrophil-to-lymphocyte ratio and systemic immune-inflammation index-biomarkers in interstitial lung disease. Medicina (Kaunas). (2020) 56:381. doi: 10.3390/medicina56080381, 32751302 PMC7466218

[25] Sargin G Baris K Gulen ST. Systemic immune-inflammation index in the evaluation of Sjogren's syndrome associated with interstitial lung disease, interstitial pneumonia with autoimmune features, and idiopathic pulmonary fibrosis. Adv Med Sci. (2025) 70:57–61. doi: 10.1016/j.advms.2024.12.001, 39675699

[26] Xing H Liang H. The clinical value of KL-6 for predicting the occurrence and severity of connective tissue disease-associated interstitial lung disease is not affected by CTD type or treatment. PeerJ. (2024) 12:e17792. doi: 10.7717/peerj.17792, 39131623 PMC11317038

[27] Ecesoy V Ecesoy H. Assessment of new inflammatory indexes in systemic sclerosis with interstitial lung disease. Eurasian J Med. (2025) 57:1–7. doi: 10.5152/eurasianjmed.2025.251103, 41342238 PMC12621640

[28] Huang L Li X Zhou W Zhu H Lao Y Huang X . The clinical value of the neutrophil-to-lymphocyte ratio, the C-reactive protein-to-albumin ratio, the systemic inflammatory index, and the systemic inflammatory response index in patients with the anti-synthetase syndrome. J Inflamm Res. (2024) 17:3617–28. doi: 10.2147/JIR.S460610, 38855168 PMC11162194

[29] Yu C Liu B Liu Y Yuan Y Zhakeerjiang X Wei C . The clinical values of laboratory inflammatory and composite indices in predicting rapidly progressive interstitial lung disease and prognosis in anti-MDA5 dermatomyositis patients. Clin Rheumatol. (2026) 45:1129–1140. doi: 10.1007/s10067-025-07923-w, 41537946

[30] Tan D Shang Y Wang J Sheng N. Predictive role of inflammatory markers in characterizing the progression of interstitial lung disease: a retrospective analysis. Am J Transl Res. (2025) 17:3485–95. doi: 10.62347/JZTW5621, 40535665 PMC12170371

[31] Althobiani MA Russell AM Jacob J Ranjan Y Folarin AA Hurst JR . Interstitial lung disease: a review of classification, etiology, epidemiology, clinical diagnosis, pharmacological and non-pharmacological treatment. Front Med. (2024) 11:1296890. doi: 10.3389/fmed.2024.1296890, 38698783 PMC11063378

[32] Kamiya M Carter H Espindola MS Doyle TJ Lee JS Merriam LT . Immune mechanisms in fibrotic interstitial lung disease. Cell. (2024) 187:3506–30. doi: 10.1016/j.cell.2024.05.015, 38996486 PMC11246539

[33] Cerro Chiang G Parimon T. Understanding interstitial lung diseases associated with connective tissue disease (CTD-ILD): genetics, cellular pathophysiology, and biologic drivers. Int J Mol Sci. (2023) 24:2405. doi: 10.3390/ijms24032405, 36768729 PMC9917355

